# Effect of Hole Diameter on Failure Load and Deformation Modes in Axially Compressed CFRP Laminates

**DOI:** 10.3390/ma18153452

**Published:** 2025-07-23

**Authors:** Pawel Wysmulski

**Affiliations:** Department Machine Design and Mechatronics, Faculty of Mechanical Engineering, Lublin University of Technology, 20-618 Lublin, Poland; p.wysmulski@pollub.pl

**Keywords:** CFRP composites, thin-walled plate, open-hole structures, composite failure, digital image correlation (DIC), post-critical behavior

## Abstract

This study presents a detailed analysis of the influence of hole presence and size on the behavior of CFRP composite plates subjected to axial compression. The plates were manufactured by an autoclave method from eight-ply laminate in a symmetrical fiber arrangement [45°/−45°/90°/0°_2_/90°/−45°/45°]. Four central hole plates of 0 mm (reference), 2 mm, 4 mm, and 8 mm in diameter were analyzed. Tests were conducted using a Cometech universal testing machine in combination with the ARAMIS digital image correlation (DIC) system, enabling the non-contact measurement of real-time displacements and local deformations in the region of interest. The novel feature of this work was its dual use of independent measurement methods—machine-based and DIC-based—allowing for the assessment of boundary condition effects and grip slippage on failure load accuracy. The experiments were carried out until complete structural failure, enabling a post-critical analysis of material behavior and failure modes for different geometric configurations. The study investigated load–deflection and load–shortening curves, failure mechanisms, and ultimate loads. The results showed that the presence of a hole leads to localized deformation, a change in the failure mode, and a nonlinear reduction in load-carrying capacity—by approximately 30% for the largest hole. These findings provide complementary data for the design of thin-walled composite components with technological openings and serve as a robust reference for numerical model validation.

## 1. Introduction

Composite structures have become indispensable in many areas of modern technology and everyday life. Their unique combination of favorable mechanical properties, low weight, and biocompatibility makes them particularly valuable in industries such as aerospace [1,2], automotive [3,4], construction [5], and medicine [6,7,8,9]. In medical applications, for instance, composites are used in the fabrication of implants, prostheses, and devices supporting tissue regeneration. Among these materials, carbon fiber-reinforced polymers (CFRPs) have emerged as a key alternative to traditional isotropic materials. Thanks to their high strength-to-weight ratio and resistance to variable loading conditions, CFRP laminates are increasingly used in demanding engineering applications where both mechanical performance and weight savings are critical.

One of the common structural design challenges involves the necessity of introducing technological openings, which serve mounting, maintenance, or structural purposes [10,11,12]. These openings introduce disturbances in the stress distribution, which can significantly affect the load-bearing capacity and stability of the component, particularly under service load conditions [13]. The issue of structural weakening due to holes has been extensively investigated in the scientific literature, both experimentally and numerically [14,15]. Takamoto et al. [16] conducted studies on thin-ply CFRP laminates with holes, analyzing the effect of ply thickness and stacking sequence on compressive strength using Hashin failure criteria and the cohesive zone model. Gupta et al. [17], in their literature review, demonstrated that the size, shape, and position of a hole significantly influence the damage distribution and strength of FRP laminates [18,19,20].

In the context of failure mechanisms, Suemasu et al. [21] demonstrated that the dominant damage initiation mechanism in quasi-isotropic laminates with an open hole is the weakening of the 0° plies. Wang et al. [22] identified the influence of the clustering effect on the development of microcracks and local buckling. The issue of interlaminar quality and its impact on failure was investigated by Xiao et al. [15], who showed that poor adhesion can accelerate delamination around the hole.

Of particular note is the micromechanical approach to fatigue life prediction presented by Cai et al. [23], as well as the analysis of complex hole configurations by Ma and Huang [24]. In the field of progressive damage analysis, important contributions were made by Khedkar and Chinthapenta [25], while Guynn and Bradley [26] provided one of the first comprehensive treatments of micromechanical failure mechanisms in the vicinity of a hole, including local buckling and fiber collapse.

Although this study is experimental in nature, the findings provide a critical benchmark for validating advanced computational models of failure in fiber-reinforced composites. Recent developments in the field of composite mechanics increasingly rely on high-fidelity numerical simulations to capture both local and global damage evolution in anisotropic laminates. Approaches such as progressive failure analysis (PFA) [27], cohesive zone models (CZMs) [28,29], and phase-field methods for fracture have proven effective in simulating delamination, matrix cracking, and fiber breakage under complex loading scenarios. The recent work by De Maio et al. [30] applies a coupled phase-field and cohesive modeling framework to predict crack propagation in layered composite structures, capturing interactions between intralaminar and interlaminar failures. Similarly, Duan et al. [31] demonstrate the effectiveness of CZM in modeling open-hole compression failure, closely aligning with experimental trends in load–shortening and load–deflection responses. The complexity of failure in laminated composites with an open hole arises from the interaction of multiple mechanisms, including fiber microcracking, delamination [16,17,18], matrix cracking [14,32], and local buckling, particularly in the 0° plies. Currently, advanced failure models are employed, such as Hashin criteria, cohesive zone models, and progressive damage analysis [33,34,35]. Williamson and Thatcher [36] point out that under biaxial loading conditions, CFRP laminates with an open hole exhibit diverse failure paths depending on the anisotropy of the ply configuration.

The stability of axially loaded thin-walled CFRP plates is a particularly critical issue in the context of aerospace and space structures [37,38,39]. Studies have shown that the presence of an opening significantly affects the buckling behavior [40] and can lead to accelerated damage, ultimately resulting in a complete loss of load-bearing capacity of the composite structure [41]. Loughlan [42] investigated the behavior of thin-walled composite plates under bending and torsion, demonstrating significant variability in stability levels depending on the type of loading and hole geometry [36,40].

The load-bearing capacity and buckling resistance of composite laminates are also influenced by the manufacturing technology (autoclave, RTM, and prepreg) and the stacking sequence of the laminate [43,44,45]. The use of thin-ply laminates improves resistance to microcrack initiation and may enhance structural performance in the presence of an opening. Falkowicz and Rozylo [46] demonstrated that an appropriate arrangement of asymmetric layers can increase the buckling resistance of plates with holes.

Based on the aforementioned studies, the present research focuses on the analysis of the full mechanical response of CFRP composite plates with circular holes of 2, 4, and 8 mm in diameter. The plates were manufactured using autoclave technology from an eight-ply symmetric carbon/epoxy laminate and had dimensions of 100 × 20 × 1.048 mm. They were subjected to axial compression up to total structural failure, with force and axial shortening recorded throughout the loading process. A non-contact optical measurement system (Aramis DIC) was employed to capture transverse deflections and identify buckling modes. Particular emphasis was placed on analyzing the failure modes and their correlation with the hole geometry. The novelty of this study lies in the simultaneous use of two independent measurement methods—global machine displacement and local digital image correlation (DIC)—to analyze post-critical behavior of CFRP plates with varying hole diameters under axial compression. This dual approach enables accurate characterization of boundary effects and localized deformation phenomena, particularly in the failure phase, which is rarely addressed in prior experimental works. The findings are particularly relevant for high-performance structural components in aerospace, automotive, and civil engineering applications, where thin-walled composite elements with technological openings must sustain complex compressive loads without premature failure. The results provide valuable reference data for the design of thin-walled composite components and for the validation of numerical FEM models [47,48,49].

## 2. Test Specimen and Methodology

The object of the study was a rectangular carbon fiber-reinforced polymer (CFRP) plate with a central through-hole [50]. The material, laminate layup, dimensions, and diameters of the through-holes were selected based on the literature review—see Table 1. The reviewed papers are mainly concerned with the study of tensile laminate structures. Based on the reviewed studies, the parameters of the specimens were selected, and it was decided to carry out experimental studies of compressed composite plates weakened by a hole. This was to fill the gap in the literature regarding the description of the effect of a hole on the behavior of composite structures over the full load range using the state-of-the-art Aramis optical non-contact measurement method. The selection of the final parameters of the test samples was influenced by scaling effects and production constraints.

**Figure 1:**
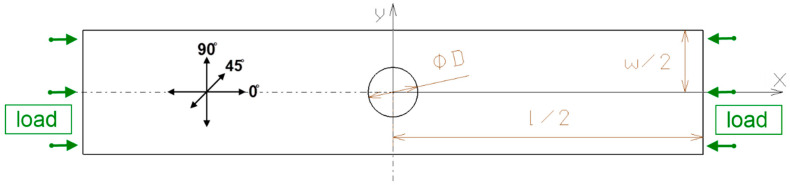
Schematic of the CFRP plate with fiber orientation and loading method.

A commonly used, symmetric laminate stacking sequence relative to the mid-plane was selected for the tests: [45°/−45°/90°/0°_2_/90°/−45°/45°]. The laminate consisted of eight plies, each with a nominal thickness of 0.131 mm, resulting in a total plate thickness of t = 1.048 mm. The effective test area of the plate measured l = 100 mm in length and w = 20 mm in width (Figure 1). A through-hole of diameter D = 2 mm, 4 mm, or 8 mm was machined precisely in the center of the specimen test area (coordinate point xy = [0, 0]) following the geometry presented in Figure 1.

**Figure 2:**
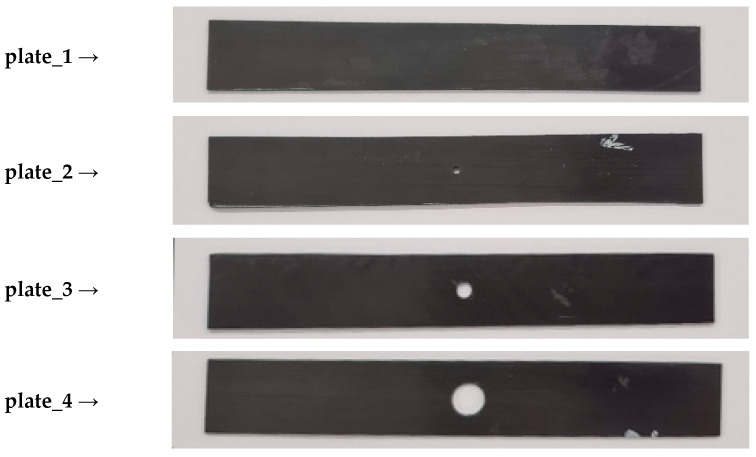
Set of real test specimens.

The test specimens were fabricated from carbon/epoxy prepreg tape (HexPly system, Hexcel, Stamford, CT, USA). The composite laminates were manufactured using autoclave processing. The manufacturing parameters included a vacuum pressure of 0.08 MPa applied to the layup, an autoclave overpressure of 0.4 MPa, a curing temperature of 135 °C, and a total processing time of 2 h. After curing, the composite structure was cut into specimens with nominal dimensions of 100 mm × 20 mm. Through-holes of 2 mm, 4 mm, and 8 mm diameter were precisely drilled at the center of each plate. The prepared test specimens are presented in Figure 2.

**Figure 3:**
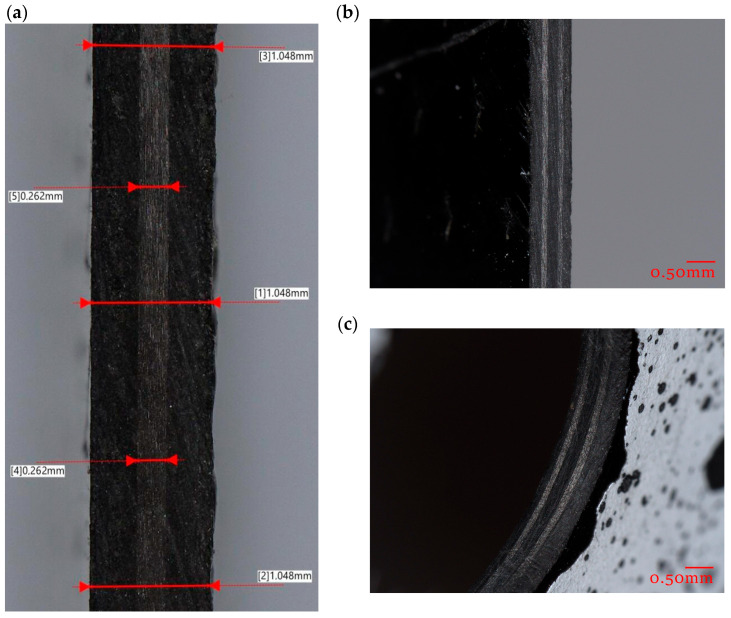
Microscopic verification of sample quality: (**a**) longitudinal view through the thickness, (**b**) transverse view through the thickness, (**c**) view of through-hole.

The manufacturing quality of the test specimens was evaluated using microscopic analysis. Figure 3a presents a longitudinal cross-section of the specimen. In this view, dimensional measurements were conducted, including total laminate thickness at three locations and the thickness of the central 0°_2_ ply at two points. Figure 3b shows a transverse cross-sectional view, where the fiber distribution across the laminate layers is clearly visible. The quality of the drilled through-hole is also illustrated in Figure 3. It should be noted that all specimens were cut with high precision.

**Figure 4:**
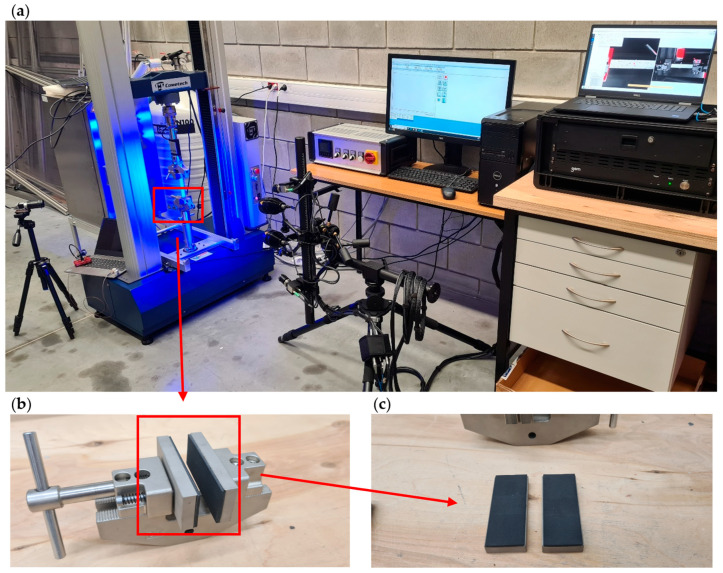
Experimental setup used for compression testing of CFRP specimens with a central hole: (**a**) integrated system consisting of a universal testing machine (UTM) and ARAMIS digital image correlation (DIC) system for full-field strain and displacement measurements, (**b**) testing machine grip, and (**c**) anti-slip clamps.

Experimental compression tests on CFRP plates with a central through-hole were carried out using a Cometech universal testing machine (UTM) equipped with a 2.5 kN load cell. The loading was displacement-controlled, with a constant crosshead speed of 1 mm/min. All compression tests were conducted under standard laboratory conditions at an ambient temperature of 22 ± 1 °C and relative humidity of 40–50%. These parameters were monitored throughout the testing campaign to ensure consistency and to minimize the influence of environmental factors on the mechanical behavior of the CFRP specimens. Given the sensitivity of polymer matrix composites to temperature and moisture, testing under stable conditions was essential for obtaining reliable and reproducible results. Each specimen was clamped at both ends to a depth of 20 mm, leaving an effective test section of 100 mm × 20 mm, with the hole positioned precisely at the center. Special anti-slip clamps were used to ensure proper boundary conditions and to eliminate plate slippage in the clamps, as shown in Figure 4b,c. The tests were conducted until the complete structural failure of the composite plate, i.e., full loss of load-carrying capacity [55,56,57]. Throughout the loading process, compressive load and axial displacement were continuously recorded as a function of time, enabling the determination of the full load–displacement response of the structure.

**Figure 5:**
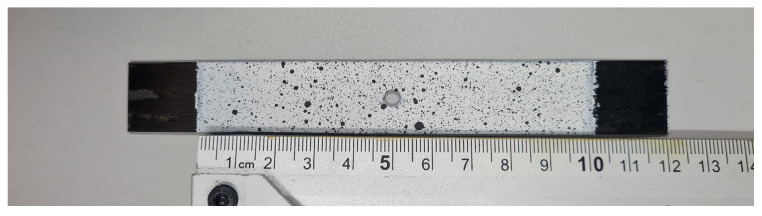
Painted sample with high-contrast speckle pattern.

To capture the detailed deformation behavior of the tested specimens, an advanced non-contact measurement system—ARAMIS by ZEISS (Oberkochen, Germany)—was employed. This system is based on the digital image correlation (DIC) technique and consists of two synchronized high-resolution cameras mounted on an adjustable tripod, enabling the accurate reconstruction of full-field displacement and strain distributions on the specimen surface. Prior to testing, the surface of each specimen was coated with a high-contrast speckle pattern, ensuring the reliable tracking of surface geometry changes throughout the loading process—see Figure 5. The optical measurement system used was configured specifically for this type of study to record the deformation of the compressed slab to the best possible extent. Each camera had a high resolution of 4096 px × 3000 px. This allowed 12 Mpx images to be taken at up to 25 Hz. The Aramis system uses two cameras to measure 3D displacements and deformations of the object under test without contact, based on digital image correlation technology. The two cameras capture images of the object’s surface, and then, the software analyzes the differences in the images to determine the 3D displacements and deformations.

Operating principle:

- Calibration: The system is calibrated to determine the geometric parameters of the two cameras and their relative positions.
- Lighting: The surface of the object under investigation is illuminated and covered with a random pattern that facilitates the tracking of changes.
- Image registration: Two cameras take a series of images of the object under study at a set frequency. Each camera captures the images in its own perspective.
- Image correlation: The software analyzes the images from the two cameras, identifying the same points on the surface of the object in successive images. It then compares their positions in 3D space.
- Calculation of displacements and deformations: Based on the differences in the positions of the points, the system calculates the 3D displacements and deformations at each point on the object surface.
- Data analysis: The results of the measurements are presented in the form of deformation maps, which allow the analysis of the behavior of the material under load.

The integration of the DIC system with the universal testing machine made it possible to acquire comprehensive data on the behavior of CFRP plates weakened by a central hole—both in terms of global axial displacement (load–shortening characteristics) and local out-of-plane deformations (load–deflection characteristics). This approach enabled the precise identification of deformation and damage mechanisms in the post-critical state, up to the complete structural failure of the composite material. A complete view of the experimental setup, including the specimen mounted in the UTM and the integrated DIC measurement system, is shown in Figure 4.

A total of four CFRP specimens were analyzed in the study—each representing a different dimension of hole diameter (0 mm, 2 mm, 4 mm, and 8 mm). The present work is part of a larger experimental and numerical study on the behavior of open-hole laminate, in which 22 autoclave-processed CFRP sheets were produced and tested under various loading conditions, including tension and compression [14,40,50,58]. In the authors’ previous work, 3 to 12 specimens were tested to ensure statistical repeatability and validation of FEM models. Based on these tests, the influence of boundary conditions—especially clamp slippage and localized strain anomalies—was identified as a critical load in the assessment of the post-critical load. Based on this, it was decided to carry out a detailed comparison of deformation modes on four geometrically different plates, with a focus on validating two methods. It is worth noting that the production of CFRP specimens using autoclave curing involves significant costs and production constraints. Given the research team’s extensive knowledge of composite testing and numerical validation, the approach adopted offers a high-resolution, cost-effective methodology for investigating failure phenomena in open-hole laminates.

## 3. Results and Discussion

The experimental investigations aimed to evaluate the effect of central holes of varying diameters on the structural behavior of CFRP (carbon fiber-reinforced polymer) laminates with a stacking sequence of [45°/−45°/90°/0°_2_/90°/−45°/45°] under axial compression. The loading was applied until complete structural failure occurred. The study focused on analyzing deformation modes, failure mechanisms, and displacement characteristics, including load–deflection and load–shortening responses of specimens with and without holes.

The initial critical buckling load (P_cr_) for the CFRP plate analyzed in this study was previously determined both experimentally and numerically in [40]. The earlier work, which used linear finite element buckling analysis and laboratory tests using identical material, layer arrangement, and geometry, established a verified critical load. In the present study, we build on these findings by extending the analysis to the post-critical state, with a particular focus on nonlinear strain development, load redistribution, and damage propagation. These aspects—especially local failure mechanisms dependent on the hole size—are not easily captured by classical analytical methods and require high-resolution experimental techniques such as digital image correlation (DIC). The dual measurement strategy used here provides insight into the degradation of the structure under compressive loading, offering an important complement to previously reported critical state data.

**Figure 6:**
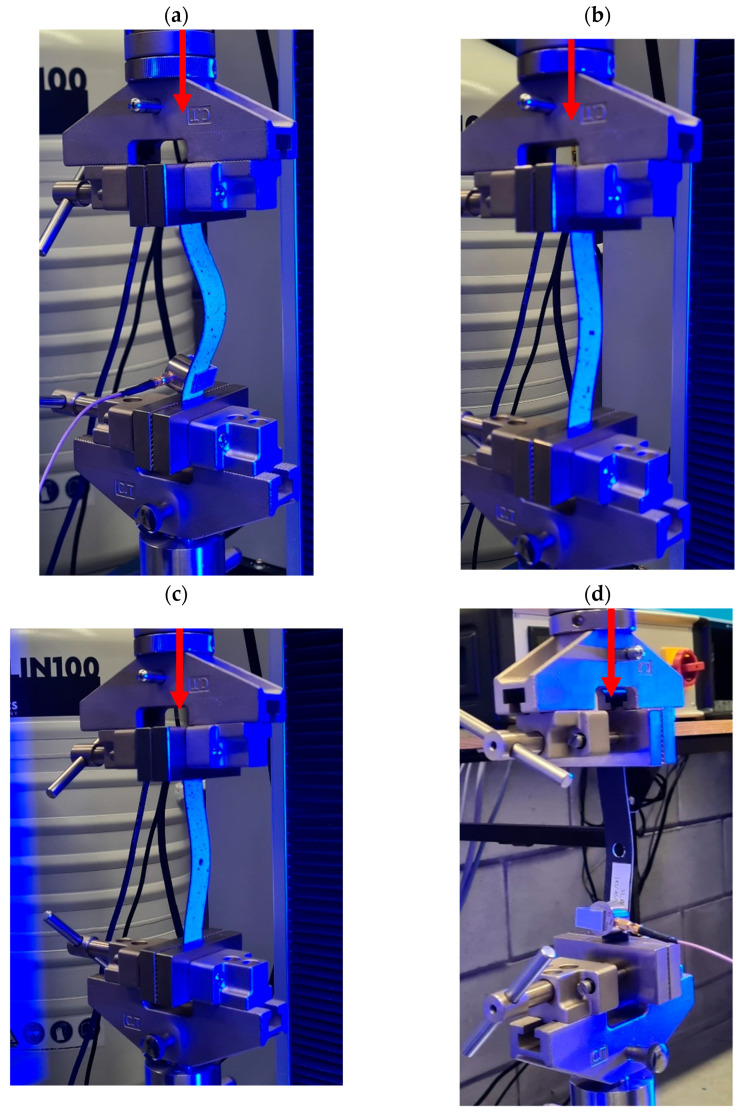
Final deformation state of the compressed plate: (**a**) plate_1, (**b**) plate_2, (**c**) plate_3, (**d**) plate_4.

Figure 6 presents a sequence of images documenting the post-critical deformation behavior of the tested CFRP plates, i.e., beyond the onset of buckling, and illustrates their final failure modes under axial compressive loading. The reference plate without a hole (Figure 6a) exhibited a uniform buckling pattern, forming a single half-wave deformation [40]. However, the presence of a central hole significantly altered the deformation behavior. In the specimen with a 2 mm hole (Figure 6b), displacement localization was observed around the hole, with the premature initiation of local buckling. Increasing the hole diameter to 4 mm (Figure 6c) resulted in more pronounced asymmetric deformation. The largest hole (8 mm, Figure 6d) caused highly localized buckling, which may accelerate structural degradation and lead to earlier failure.

The centrally located hole significantly disturbs the uniform stress distribution and the behavior of the plate, acting as a damage initiator. In the post-critical state, a clear localization of deformation is observed around the hole—the plate surface deflects asymmetrically, forming regions of high curvature that indicate developing local buckling and material warping. The plate deformation is neither global nor symmetric but concentrated and local, which is typical for elements weakened by a hole. The material around the hole experiences localized bulging, with deformations concentrated in its immediate vicinity.

The anisotropy of the laminate stacking sequence [45°/−45°/90°/0°_2_/90°/−45°/45°] provides relative in-plane stiffness; however, in the through-thickness direction—especially in the presence of a hole—this results in a reduction in laminate stiffness, particularly in the ±45° plies, which are highly sensitive to shear. Plate deflections are observed, which may indicate the onset of interlaminar delamination in the later stages of damage progression. The hole introduces disruptions in fiber continuity, especially in the 0° and 90° plies, which are critical for carrying axial and transverse stresses. This explains the decrease in shortening and deflection with increasing hole diameter in the composite plate.

Subsequent stages of increasing deflection indicate the development of nonlinear deformation. The specimen deforms locally and asymmetrically, suggesting low post-critical stiffness that leads to the rapid amplification of the deformation and ultimately to the failure of the composite structure.

**Figure 7:**
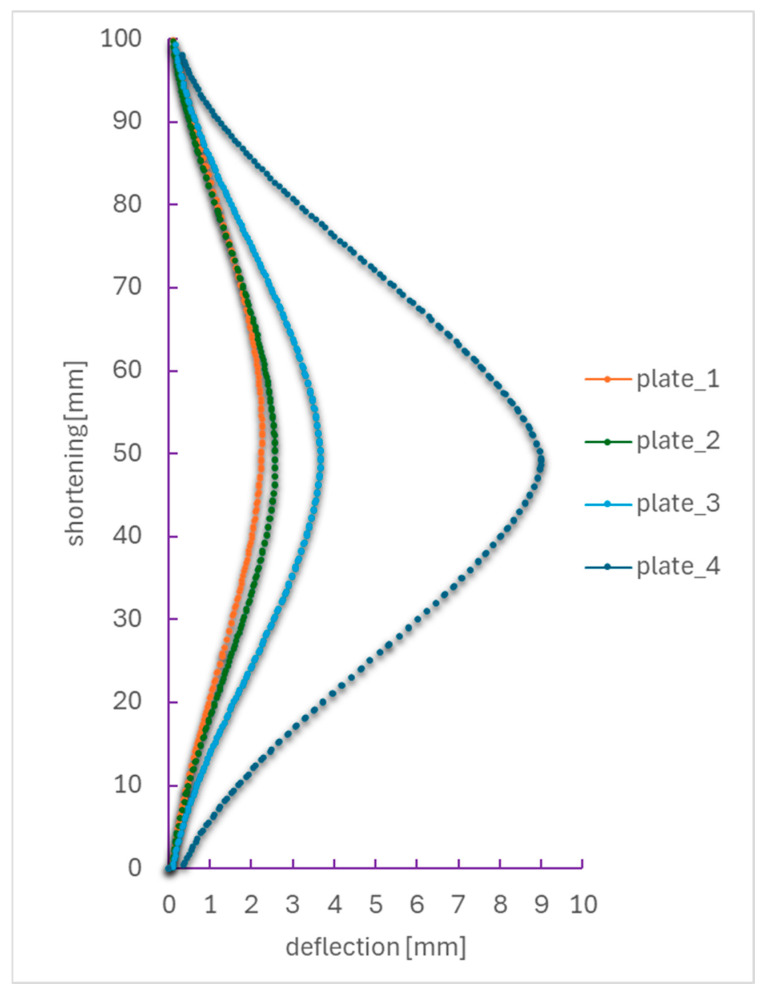
Deflection–shortening relationship of compressed plates in 174 N load (max load plate_4).

For a more comprehensive analysis of post-critical deformation, the Aramis digital image correlation (DIC) system was employed, enabling the precise determination of the relationship between specimen shortening (vertical axis) and its transverse deflection (horizontal axis). In order to compare the load response of the plates under the same loading conditions, the paths were determined for a load of 174 N. This is the maximum load achieved for the weakest sample, plate_4. Figure 7 presents a comparison of results for four plates with different geometries: plate_1—reference plate without a hole (orange), plate_2—hole diameter of 2 mm (green), plate_3—hole diameter of 4 mm (light blue), and plate_4—hole diameter of 8 mm (dark blue). All curves exhibit a nonlinear deformation behavior typical for the deep post-critical state, with deflection increasing significantly at relatively small increments of shortening. This indicates that the plate transitioned from a membrane state to a bending-dominated state.

The quantitative analysis of the compression behavior of the specimens is presented in Table 2, which shows the relationship between lateral deflection and axial shortening for all cases. The plate without a hole (plate_1) achieves the smallest deflection (~2.25 mm) with the smallest possible shortening (~0.19 mm), indicating a wide range of stable coverage response. The introduction of a 2 mm diameter hole (plate_2) resulted in a slight increase in both maximum shortening and deflection, indicating the onset of stiffness degradation. When the hole diameter was increased to 4 mm (plate_3), a significant increase in both deflection (~3.67 mm) and shortening (~0.35 mm) was observed. This behavior suggests a transition to more localized deformation associated with stress concentration around the edge of the hole. The greatest degradation was observed for the 8 mm hole (plate_4), where the structure weakened prematurely, reaching a maximum deflection of about 9 mm and a shortening of about 2 mm. The results clearly indicate a strong relationship between hole diameter and the structural integrity of the composite under compressive loading.

In the next stage of the analysis, the focus was placed on comparing the load–shortening paths, which describe the relationship between the compressive load and the specimen shortening. These curves were determined independently using two methods:

- Testing machine—shortening was determined based on the displacement of the machine’s upper crosshead (global displacement), which includes the total movement of the grips but does not account for local deformation effects of the specimen within the grips or possible slips.
- ARAMIS system (DIC)—shortening was measured directly in the specimen’s measurement area (20 × 100 mm) using the digital image correlation (DIC) method, allowing for the capture of the actual local material shortening within the analyzed region.

**Figure 8:**
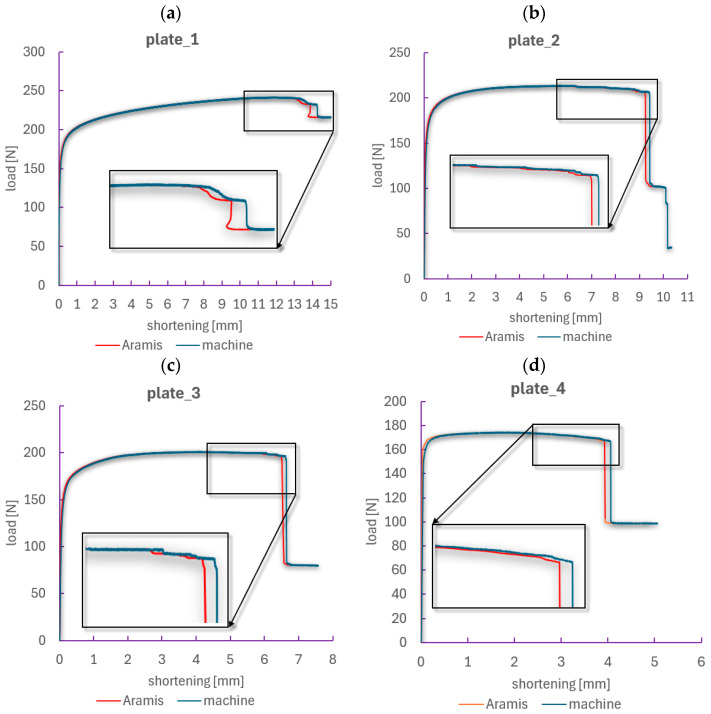
Validation of load–shortening paths: (**a**) plate_1 (no hole), (**b**) plate_2 (2 mm hole), (**c**) plate_3 (4 mm hole), (**d**) plate_4 (8 mm hole).

Figure 8 presents comparative plots of both methods for each specimen. The graphs also include zoomed-in views of the regions where the curves begin to diverge, thereby facilitating the analysis of the differences.

In the initial loading phase, up to the maximum load, both methods show a high level of agreement—covering the critical and post-critical states. This results from the absence of significant local deformations and negligible influence of boundary conditions, reflecting good interaction between the specimen and the machine grips. However, the nature of the compared load–shortening curves changes in the deep post-critical state, near the phase of composite structure failure, where the curves begin to diverge. For each plate, differences in the recorded shortening are evident—measurements from the testing machine indicate failure at greater shortening values than those from the ARAMIS system. This discrepancy is due to uncontrolled slips in the grips or deformations occurring outside the ARAMIS measurement area.

In the case of plate_1 (without a hole), the differences between the methods are relatively small—the structure is stable, and the deformation develops gradually (Figure 8a). For plate_2 and plate_3 (holes of 2 and 4 mm), initial sudden changes in the slope of the curves are observed, corresponding to the onset of local damage around the hole. Differences between the methods become more noticeable, especially in the final stage of the test. The largest discrepancies were noted for plate_4 (8 mm hole), where the composite structure undergoes rapid failure, and the deformations are highly localized near the hole—the ARAMIS curve shows a sudden drop in load at smaller shortening compared to the data from the testing machine.

The application of two methods for determining load–shortening paths enabled a more accurate understanding of the phenomena accompanying the compression of CFRP composite plates. Although data from the testing machine are valuable, they may not fully reflect the actual material behavior in the region of interest, especially after reaching the maximum load. In contrast, the ARAMIS method, based on local measurements, provides more precise information about the actual shortening and deformations of the specimen. This comparison demonstrates that for an accurate assessment of composite structural behavior—particularly in the post-critical phase—the use of an optical, non-contact measurement system is essential. The DIC method can serve as an effective tool for validating numerical models and analyzing degradation in composite materials [14,58].

In the final stage of the experimental study, the failure modes of CFRP composite laminate plates with a [45°/−45°/90°/0°_2_/90°/−45°/45°] layup were analyzed, with particular emphasis on the influence of the hole on the failure mode of the hole-weakened composite plate. The specimens were subjected to axial compression until the complete loss of load-bearing capacity and physical structural failure.

**Figure 9:**
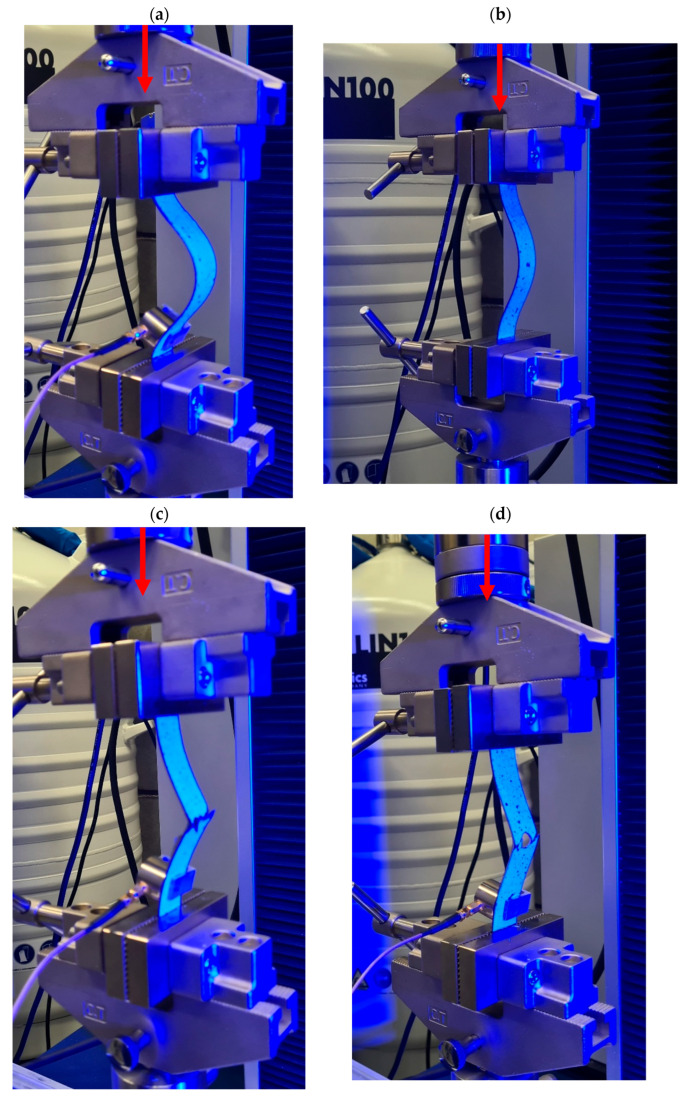
Failure mode analysis of hole-weakened composite plates: (**a**) plate_1—plate without a hole (reference structure), (**b**) plate_2—plate with a 2 mm hole, (**c**) plate_3—plate with a 4 mm hole, and (**d**) plate_4—plate with an 8 mm hole.

Figure 9 shows the final failure state of each analyzed plate. For plate_1, failure was characterized by regular deformation, with damage gradually developing in the central part of the plate—see Figure 9a. The cracking mechanism involved delaminations and local damage to the matrix and fibers in the region of maximum deflection. In the case of plate_2, initial signs of damage localization appeared directly around the hole—see Figure 9b. The hole acted as a stress concentrator, and damage developed around its edges. Failure occurred due to the combination of local damage and global buckling. The failure mode of plate_3 was more localized and asymmetric—see Figure 9c. The hole significantly altered the damage propagation path. Characteristic delaminations and layer separations along the fiber direction at a 45° angle were observed, typical for shear loading in laminates. For plate_4, the dominant failure mechanism was sudden, localized damage with crack concentration around the hole, propagating transversely through the laminate thickness.

Although the failure analysis presented in this study is based primarily on visual and macroscopic observations, the identified deformation patterns suggest underlying microstructural damage mechanisms typical for CFRP laminates under compressive loads with geometric discontinuities. In particular, the localized buckling and asymmetric deformation near the hole edge—especially in specimens with 4 mm and 8 mm holes—are consistent with the onset of interlaminar delamination and matrix cracking initiated at the fiber–matrix interface. Given the laminate stacking sequence ([±45°/90°/0°_2_/90°/±45°]), failure likely initiates within the ±45° layers due to their lower in-plane shear resistance, leading to localized shear band formation. These shear-dominated zones can cause progressive fiber/matrix interface debonding, which facilitates delamination propagation between adjacent plies. Such mechanisms have been previously observed in similar open-hole compression studies (e.g., [15,21,25]), where fiber misalignment and micro-buckling near the hole trigger local collapse and layer separation. Although detailed microstructural imaging (e.g., SEM or micro-CT) was not conducted in this phase of the project, future work will include cross-sectional scanning of failed specimens to validate the presence and extent of fiber breakage, matrix cracking, and interfacial decohesion. These techniques will enable direct correlation between observed global failure patterns and the micro-mechanical damage processes responsible for structural degradation.

**Figure 10:**
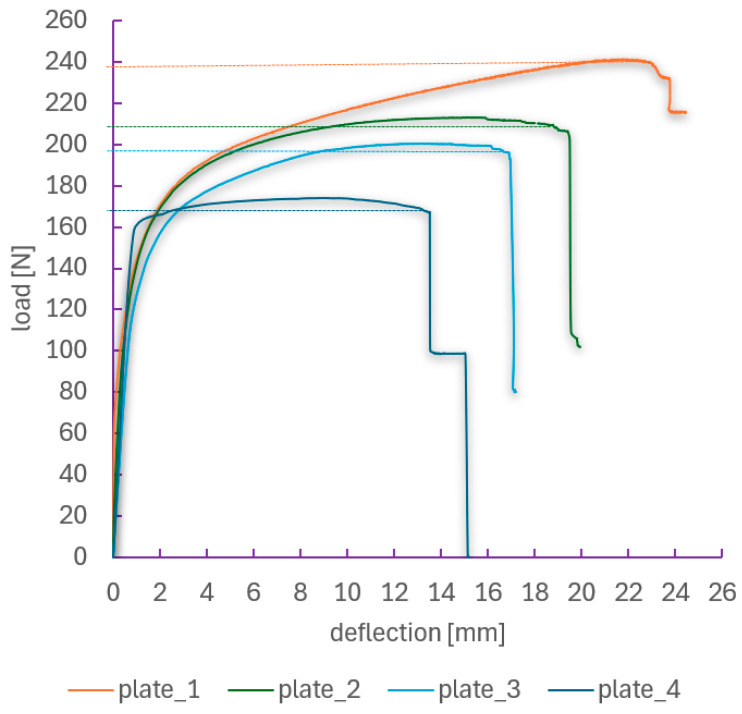
Load–deflection paths over the full loading range.

**Figure 11:**
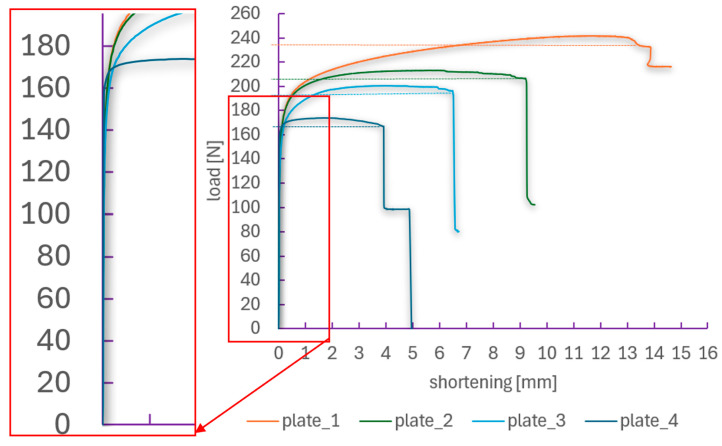
Load–shortening paths over the full load range.

The experimental analysis concluded with the determination of the failure load for the tested CFRP composite plates with the layup configuration [45°/−45°/90°/0°_2_/90°/−45°/45°], based on the results of compression tests. To this end, load–deflection and load–shortening characteristics were analyzed, as presented in Figure 10 and Figure 11, respectively. The failure loads were defined as the maximum compressive force recorded during the test prior to the sudden loss of structural load-carrying capacity.

The recorded curves illustrate the structural response over the full loading range up to the failure of plates with various hole diameters. The reference plate (plate_1, without a hole) exhibited the highest compressive strength, with a failure load of approximately 240 N. Deformation progressed gradually, featuring a distinct post-critical phase. As expected, plate_4 (with an 8 mm hole) demonstrated the lowest strength, with a failure load below 170 N and an almost immediate loss of stability after reaching peak load. All specimens show a typical response pattern: an increase in load up to the maximum, followed by a sharp drop associated with crack propagation and loss of load-bearing capacity. For plates with larger holes, earlier damage initiation and a reduced ability to accommodate post-critical deformation were observed. Despite geometric differences, the load–shortening curves for all specimens share a similar trend—after an initial phase of linear load increase (critical state), a nonlinear load increase occurs with relatively large displacements (post-critical state), followed by a sudden drop in force, indicating the failure of the composite structure.

**Figure 12:**
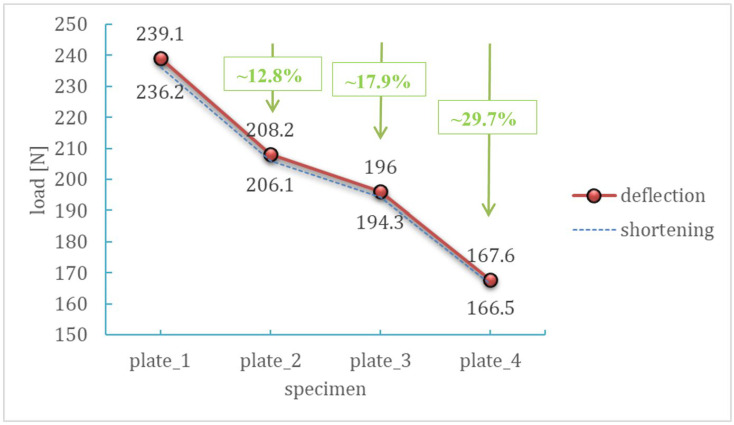
Effect of the hole on the failure load of a composite plate with a hole.

The determined failure load values are summarized in Table 3, while their dependence on hole diameter is presented graphically in Figure 12. A clear decreasing trend is visible, confirming that the presence of a hole significantly affects the structural response of the composite plate. Importantly, the reduction in load-bearing capacity is nonlinear—the most substantial changes occur for holes larger than 4 mm. The differences between the values determined from the deflection and shortening curves are very small (below 1.5%), indicating high consistency and reliability of both measurement methods.

The relationship between failure load and hole diameter is nonlinear—the most significant differences are observed between plate_3 and plate_4, despite the diameter increase being the same as in previous cases (a factor of ×2)—see Figure 12. This suggests the existence of a critical diameter range in which the reduction in load-bearing capacity becomes particularly pronounced.

The plate without a hole (plate_1) achieved the highest failure load values, making it the reference point. The average value was 237.6 N. Introducing a 2 mm hole (plate_2) resulted in a moderate reduction in load-carrying capacity—approximately 13% compared to the reference plate. Despite the presence of the hole, the plate still demonstrated a high ability to sustain compressive loads. Plate_3 (4 mm hole) exhibited a further decrease in failure load—nearly 18%—indicating a significant influence of hole diameter on the localization and development of damage in the composite structure. The largest hole—8 mm (plate_4)—caused a drastic weakening of the plate. The average failure load was only 167.1 N, representing an almost 30% reduction compared to the plate without a hole. At the same time, the load–deflection curves indicate the most abrupt failure behavior.

The presence of a central hole significantly affects the load-carrying capacity and behavior of CFRP laminates under compression. Even small holes lead to strain concentration, resulting in reduced stiffness and a change in the failure mode. Larger holes promote asymmetric and localized deformations, substantially decreasing the structural capacity. The obtained results are of great importance for the safe design of composite components in engineering applications, where the presence of holes (e.g., for fasteners) is often unavoidable.

The results obtained in this study align with known trends in the literature, which confirm that open-hole geometries reduce the load-bearing capacity of CFRP laminates. However, this work advances current knowledge in several key aspects. First, the combination of machine-based and DIC-based measurements provides a more nuanced understanding of the deformation and failure mechanisms, particularly in the deep post-critical regime. While previous studies, such as Takamoto et al. [16] and Ma & Huang [24], have examined failure under open-hole compression, they largely relied on global measurements or numerical predictions. In contrast, the present study offers detailed experimental insights into local deformation fields and validates the influence of grip boundary conditions on failure load accuracy. Moreover, the observed nonlinear relationship between hole diameter and failure load, especially the sharp drop between 4 mm and 8 mm, adds new quantitative data that can inform threshold criteria for design. This level of detail is critical for engineers modeling damage tolerance in aerospace fuselage panels, automotive crash structures, and precision composite housings in robotics.

## 4. Conclusions

The experimental results presented in this study provide new insights into the compressive behavior of CFRP laminates with central circular holes. Based on the analysis of load–shortening, load–deflection, and failure patterns, the following conclusions can be drawn:

- Hole size strongly influences failure load, with a nonlinear reduction ranging from ~13% for a 2 mm hole to nearly 30% for an 8 mm hole. The most significant drop occurs between 4 mm and 8 mm, indicating a critical geometric threshold for structural degradation.
- Deformation and failure modes evolve with hole diameter: from uniform buckling in the reference plate to asymmetric, localized damage and delamination near the hole edges in larger openings.
- Dual measurement methods—global machine displacement and local DIC—provide consistent results in the elastic and critical state but diverge in the post-critical state. DIC enables more accurate detection of local deformation, especially near failure.
- The presence of the hole alters load paths and local stiffness, triggering the earlier onset of post-buckling behavior and limiting the load-carrying capacity of the structure.
- Quantitative analysis of deflection and shortening confirms the need for localized strain monitoring in design and testing of thin-walled CFRP structures with holes.

These findings offer practical guidance for engineering design, especially in applications where geometric discontinuities are unavoidable. The experimental data also serve as a reliable reference for future numerical simulations. In follow-up work, we plan to implement progressive failure models and cohesive zone elements to simulate the post-critical phenomena observed in this study, supported by microstructural validation techniques such as SEM and micro-CT scanning.

The practical relevance of the presented findings lies in their direct applicability to the structural design and damage assessment of CFRP components featuring holes or cutouts. The quantified relationship between hole diameter and failure load provides a data-driven foundation for defining safe geometric tolerances in lightweight engineering applications. Designers can use these results to avoid critical hole sizes that induce rapid post-critical degradation, as shown between the 4 mm and 8 mm range. Furthermore, the demonstrated divergence between global and local strain measurements underscores the importance of using full-field optical methods in the evaluation of highly stressed composite areas. This is particularly relevant in high-performance structures such as aircraft fuselage frames, satellite panels, and load-bearing composite casings, where damage initiation may occur locally but affect global performance. The dual-measurement methodology also offers engineers a reliable experimental approach to validate FEM-based predictions and assess the influence of boundary conditions in structural testing.

## Figures and Tables

**Table 1:** Literature review of tested samples.

Reference	Material	Layup	Geometry l × w (mm × mm)	Hole Diameter (mm)
[51]	CFRP	[0°/±45°/90°]_s_	127 × 25.4	3.175; 6.35; 9.525
[52]	CFRP GFRP	[0°/±45°/90°]_s_ [0°/90°]_2s_	L × 30	1.5; 3; 5; 7.5; 10; 12; 15
[53]	CFRP	[45°/0/−45°/90°]_2s_	305 × 38.1	2; 3.81; 6.35; 9.55
[54]	CFRP	[0°/90°/±45°]_2s_	280 × 76	6.35; 12.7; 25.4

**Table 2:** Measured deflection and shortening values in 174 N load (max load plate_4).

Specimen	Deflection	Shortening
	[mm]	[mm]
plate_1	2.25	0.19
plate_2	2.58	0.22
plate_3	3.67	0.35
plate_4	8.99	1.73

**Table 3:** Failure loads: deflection vs. shortening.

Specimen	Hole Diameter	Failure Load—Deflection	Failure Load—Shortening	Failure Load—Average	Relative Error
	[mm]	[N]	[N]	[N]	[%]
plate_1	0	239.1	236.2	237.6	1.21
plate_2	2	208.2	206.1	207.1	1.01
plate_3	4	196	194.3	195.1	0.87
plate_4	8	167.6	166.5	167.1	0.66

## Data Availability

The original contributions presented in the study are included in the article, further inquiries can be directed to the author.
